# Biosynthesis of the polymeric gel-forming mucin MUC5B

**DOI:** 10.1152/ajplung.00046.2016

**Published:** 2016-03-18

**Authors:** Caroline Ridley, Sara Kirkham, Sally J. Williamson, C. William Davis, Philip Woodman, David J. Thornton

**Affiliations:** ^1^Wellcome Trust Centre for Cell-Matrix Research, The University of Manchester, Manchester, United Kingdom;; ^2^Faculty of Life Sciences, The University of Manchester, Manchester, United Kingdom; and; ^3^Cystic Fibrosis/Pulmonary Research and Treatment Center, University of North Carolina, Chapel Hill, North Carolina

**Keywords:** goblet cell, mucin, mucus, cystic fibrosis, von Willebrand factor

## Abstract

MUC5B is a major polymeric mucin in the airway mucus gel and is an essential component of innate defense of the respiratory epithelium. Knowledge of the synthesis and intracellular processing of MUC5B is incomplete. We investigated the molecular details of MUC5B assembly in primary human bronchial epithelial cells (HBECs) grown at an air-liquid interface (ALI). Electrophoretic and centrifugal separations of intracellular forms of MUC5B probed with antibodies specific for non-*O*-glycosylated and *O*-glycosylated forms of the mucin identified three major intracellular populations of MUC5B (non-*O*-glycosylated monomer and dimer, and *O*-glycosylated polymers). Biophysical analysis of recombinant MUC5B COOH-terminus (CT5B; D4-B-C-CK) expressed in 293-EBNA cells showed that MUC5B dimerizes by disulfide linkage. Pulse-chase studies in the HBEC ALI cultures showed that non-*O*-glycosylated MUC5B was synthesized within 20 min of metabolic labeling and *O*-glycosylated, polymeric mucin within 2 h. Radiolabeled *O*-glycosylated mucin polymers were secreted within 2 h and the majority were released by 48 h. These data indicate that MUC5B follows a similar assembly to the related glycoprotein, von Willebrand factor (vWF); however, unlike vWF the MUC5B polypeptide shows no evidence of major proteolytic processing of D-domains during the production of the mature secreted polymeric mucin in normal and cystic fibrosis (CF) primary bronchial epithelial cells. In contrast, MUC5B D-domains were modified by neutrophil elastase, a protease commonly found in CF sputum, demonstrating that proteolytic degradation of MUC5B is an extracellular event in CF sputum. These results define the pathway for synthesis of MUC5B in primary human goblet cells.

mucus provides a dynamic, multifunctional interface between tissues and the outside environment. This glycoconjugate-rich layer is an important component of innate defense and is vital for normal physiology. In the airways the properties of the gel are tailored for transport, and, in conjunction with the ciliated epithelium, mucus functions to remove pathogens, particulates, and toxins from the lung. However, accumulation of mucus, with suboptimal transport properties, is a pathological feature of airway diseases such as cystic fibrosis (CF), chronic obstructive pulmonary disease, and asthma (15, 16, 20, 26, 30).

Polymeric mucins are major determinants of mucus properties, and in the airways the mucus gel is comprised of a heterogeneous mixture of MUC5AC and MUC5B mucins (13). MUC5B is essential for mucociliary clearance from the airways (22), and alterations in the amount, glycoform, and morphology of MUC5B have been associated with airway obstruction (12, 13, 24, 26). Alterations in MUC5B assembly and processing could contribute to the aberrant properties of mucus in disease. For example, cross-linked MUC5B mucin networks responsible for the mucus plugging the airways of an asthmatic individual have been proposed to arise from inadequate proteolytic processing, either pre- or postsecretion (24).

The synthesis of MUC5B is a complex process involving disulfide bond-mediated polymerization and extensive *O*-glycosylation. MUC5B assembles intracellularly as a linear polymer and shares features of its assembly with the related glycoprotein von Willebrand factor (vWF) (23, 34). Although not yet formally shown, as vWF, MUC5B likely forms COOH-terminal dimers in the endoplasmic reticulum, which then transit to the Golgi and are *O*-glycosylated. As with vWF, subsequent polymer formation occurs via disulfide linkage between NH_2_-terminal D3 domains (18).

Both MUC5B and vWF are large polymeric, high-molecular-weight glycoproteins that can be packaged, via a reversible calcium-dependent process, in a highly condensed state in secretory granules prior to their release (10, 11, 18). Whereas proteolytic cleavage of the vWF polypeptide has been shown to be a key step for its efficient packaging and subsequent unfolding following secretion (10), for MUC5B, proteolytic processing has only been proposed as part of the mechanism controlling the transition from the condensed packaged mucin to its expanded linear conformation in mucus (11). In vWF, following transport to the Golgi, the vWF polypeptide undergoes proteolytic processing between the D1–D2 and D′–D3 domains in the NH_2_-terminus (23). This proteolytic cleavage event releases a portion of the vWF protein consisting of the D1–D2 domains, which remains associated with the mature subunit due to calcium-dependent interaction under low pH during packaging. Upon secretion of vWF, a change in pH to 7.4 causes release of the D1–D2 “fragment” from the mature subunit, allowing extension of vWF into its correct conformation (10). Whether this mechanism is active during MUC5B synthesis remains to be shown. Analysis of CF sputum has demonstrated proteolytic modification of MUC5B (6, 9). Therefore it remains an open question as to whether MUC5B is proteolytically processed as part of its assembly, and investigation of MUC5B biosynthesis is vital for a greater understanding of mucus barrier function and dysfunction.

Studies on the native macromolecules, performed in physiologically relevant cell culture systems, are required for a more complete understanding of polymeric mucin assembly to emerge. Toward this goal we have previously characterized the biosynthetic pathway of MUC5AC mucins produced by transformed intestinal epithelial cells in culture; however, currently there are few reports on the biosynthesis of MUC5B (25, 33). Therefore, we used cultured primary human bronchial epithelial cells (HBECs), grown at an air-liquid interface (ALI), to study the biosynthesis, to study the biosynthesis of the major airway mucin MUC5B. This culture system is a well-characterized in vitro model for the airway surface epithelium that forms a confluent fully differentiated ciliated monolayer and produces MUC5B and to a lesser extent MUC5AC mucins (2, 8, 28).

To gain insight into the molecular detail of MUC5B biosynthesis we investigated *1*) the time course of MUC5B intracellular assembly, *2*) the role of COOH-terminal protein domains in the assembly process, and *3*) whether proteolytic processing was a feature of MUC5B assembly, packaging, or expansion in normal and CF cells.

## EXPERIMENTAL PROCEDURES

### 

#### Cell culture.

Normal HBECs (donor numbers 7152 and 2F1578) grown at the University of Manchester were purchased from BioWhittaker (Verviers, Belgium) and grown by methods based on those of Gray and coworkers (5). Initial expansion of cells was on Vitrogen (Nutacon, Leimuiden, The Netherlands)-coated plates, grown in bronchial epithelial cell growth medium plus bullet kit supplements (Clonetics, San Diego, CA). Subsequent cultures were grown on Transwell-COL inserts (Costar, Schiphol-Rijk, Netherlands) in ALI medium (Biofluidics, Rockville, MD) (2, 5, 17). At confluence (6–8 days) an ALI was formed and cells were fed basally three times a week.

Similar bronchial epithelial cell cultures derived from the lungs of CF patients (CF BECs) were grown at the University of North Carolina (UNC). The cells were derived from lungs obtained at transplantation under UNC Institutional Review Board-approved protocols consistent with all US federal and institutional requirements for informed consent and confidentiality. The cells were grown on the same Transwell-COL inserts as above, using similar cell culture protocols and media prepared in the UNC CF Research Center Cell Culture facility (2, 5, 17).

#### Collection of mucins.

At least once a week post-ALI formation, the apical surface of the cells was washed gently with 0.5 ml prewarmed PBS (one quick wash plus one 30-min wash). These washes were pooled (from 7–24 days post-ALI) and stored at 4°C; this pool is then referred to as apical secretions. Cells were lysed by the addition of 6 M guanidinium chloride (GdmCl), 5 mM *N*′-ethylmaleimide (lysis buffer). Before lysis, cells were washed (as above) and then transferred to wells containing PBS, to wash the underside of the inserts (24 mm), and subsequently transferred to wells containing 0.5 ml lysis buffer basally and 1 ml apically, and plates were then gently shaken overnight at 4°C. Lysates were analyzed by Western blotting after agarose gel electrophoresis and fractionated by density gradient centrifugation (Beckman Coulter Ti70 rotor, at 40,000 rpm 68 h 15°C, in 4 M GdmCl/CsCl at a starting density of 1.4 g/ml). Fractions from the density gradient were analyzed by immunodetection after slot blotting.

#### Pulse-chase experiments.

HBECs were used for pulse-chase experiments between 15 and 25 days post-ALI formation, because this has been found to be the period in which mucin production is at its greatest (8). The apical surface of the cells was washed (4 × 1 h washes) 24 h before the start of the pulse-chase period and again twice in the final 2 h of the “methionine-starved” period (incubation in methionine-free medium), to ensure the airway surface liquid collected had a high as possible labeled mucin content. Inserts were removed from ALI media and transferred to prewarmed PBS to wash the basal surface of the insert, this was then repeated. From the PBS, inserts were transferred to methionine-free ALI media; cells were incubated in this media for 4 h at 37°C, 5% CO_2_. Inserts were then transferred to wells containing methionine-free ALI media with [^35^S]l-methionine [3 MBq (83 μCi)/24 mm insert] and pulse labeled for 15, 20, or 30 min (stated in legend to appropriate figure) at 37°C, 5% CO_2_. At chase time, *t* = 0 inserts were again transferred to prewarmed PBS, twice, then into 10 × methionine ALI media and incubated between 10 min and 120 h at 37°C and 5% CO_2_. At given time points the apical surface of the cells was washed twice with PBS (one quick wash and then one 5-min wash). If the time point was more than 8 h, three washes were performed in the final hour. At each time point cells were lysed as above and lysates were dialyzed into 6 M urea, for electrophoresis, or detergent-free immunoprecipitation (IP) buffer (50 mM Tris·HCl, pH 7.4, containing 150 mM NaCl, 2 mM EDTA, and 0.02% sodium azide) for immunoprecipitation.

PBS washes of the apical surface of the cultures were pooled and GdmCl was added to 4 M, mixed overnight at 4°C, to ensure solubilization of all the mucins, and then dialyzed into 6 M urea. Radiolabeled molecules were separated by agarose gel electrophoresis, transferred to nitrocellulose, and subsequently detected by phosphoimaging (BAS-1800, Fujiphot Film, Tokyo, Japan).

#### Immunoprecipitation.

Cell lysates or apical washes were dialyzed into IP buffer; Triton X-100 was added to a final concentration of 1%, then mixed with Protein-A Sepharose CL-4B (10% wt/vol). After 1 h of mixing at 4°C, the solution was centrifuged and the resulting supernatant was mixed with fresh Protein A-Sepharose containing 1:100 LUM5B-13 and incubated with rotation at 4°C for 48–65 h. Three detergent-free IP buffer washes preceded elution of the bound molecules with two washes of 6 M GdmCl. The eluted solution was then subject to dialysis into 6 M urea before electrophoresis.

#### Agarose gel electrophoresis.

**Fig. 1.**
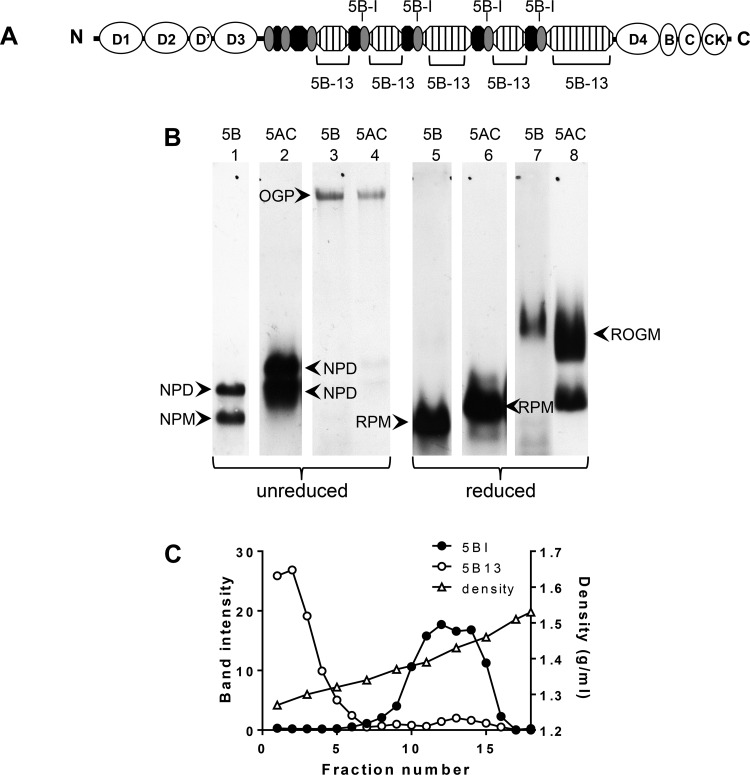
Identification of intracellular forms of MUC5B. *A*: cartoon of MUC5B NH_2_- and COOH-terminal domains (open ovals), repetitive glycosylated domains (hatched boxes), nonrepetitive glycosylated domains (solid ovals), and Cys domains (shaded ovals) highlighting the positions of the peptides used as immunogens for MAN-5BI and LUM5B-13. *B*: HBEC lysates, unreduced (*lanes 1–4*) or reduced by treatment with 10 mM DTT (*lanes 5–8*), were subjected to 0.7% (wt/vol) agarose gel electrophoresis and subsequently transferred to nitrocellulose. Western blots were probed with polyclonal antiserum LUM5B-13 (*lanes 1* and *5*), monoclonal antibody 2011 (*lanes 2* and *6*), polyclonal antiserum MAN-5BI (*lanes 3* and *7*) and polyclonal antiserum MAN-5ACI (*lanes 4* and *8*). The data presented were obtained from 2 gels and the details are given in experimental procedures. OGP, *O*-glycosylated polymeric mucin; ROGM, reduced *O*-glycosylated monomer; NPM, native polypeptide monomer; NPD, native polypeptide dimer; RPM, reduced polypeptide monomer. *C*: HBEC lysates were extracted with 6 M GdmCl and subjected to CsCl/4 M GdmCl density gradient centrifugation. After slot blotting onto nitrocellulose the non-*O*-glycosylated and *O*-glycosylated MUC5B distributions were analyzed by immunodetection with LUM5B-13 (○) and MAN-5BI (●), respectively. Fractions were also analyzed for density by weighing (△).

Briefly, samples were run on 0.7% agarose gels (wt/vol) (40 mM Tris-acetate, 1 mM EDTA pH 8, containing 0.1% SDS) for 16 h at room temperature. Where unreduced samples were run, gels were treated with reducing agent (10 mM dithiothreitol; DTT) prior to transfer. Transfer of mucins to nitrocellulose was via a vacuum blotter at 45 mbar for 1 h 30 min (25). The data presented in Fig. 1*B* were obtained from two gels with each lane equally loaded with samples of the HBEC cell lysate; one gel was loaded with unreduced samples and the other loaded with reduced samples. After electrophoresis, the mucins were transferred to nitrocellulose (see above), and before incubation with each primary antibody the membrane was sliced up into strips. Prior to use of the ECL reagent to detect the mucins, the membrane was reassembled by aligning the strips at the sample wells (marked by the dots). Different exposure times were needed because each antibody had a different affinity for its epitopes. Lanes from the blots were cut from captured digital images and assembled to yield the data presented.

#### Expression, purification, and characterization of recombinant human MUC5B COOH-terminal domains.

**Fig. 2.**
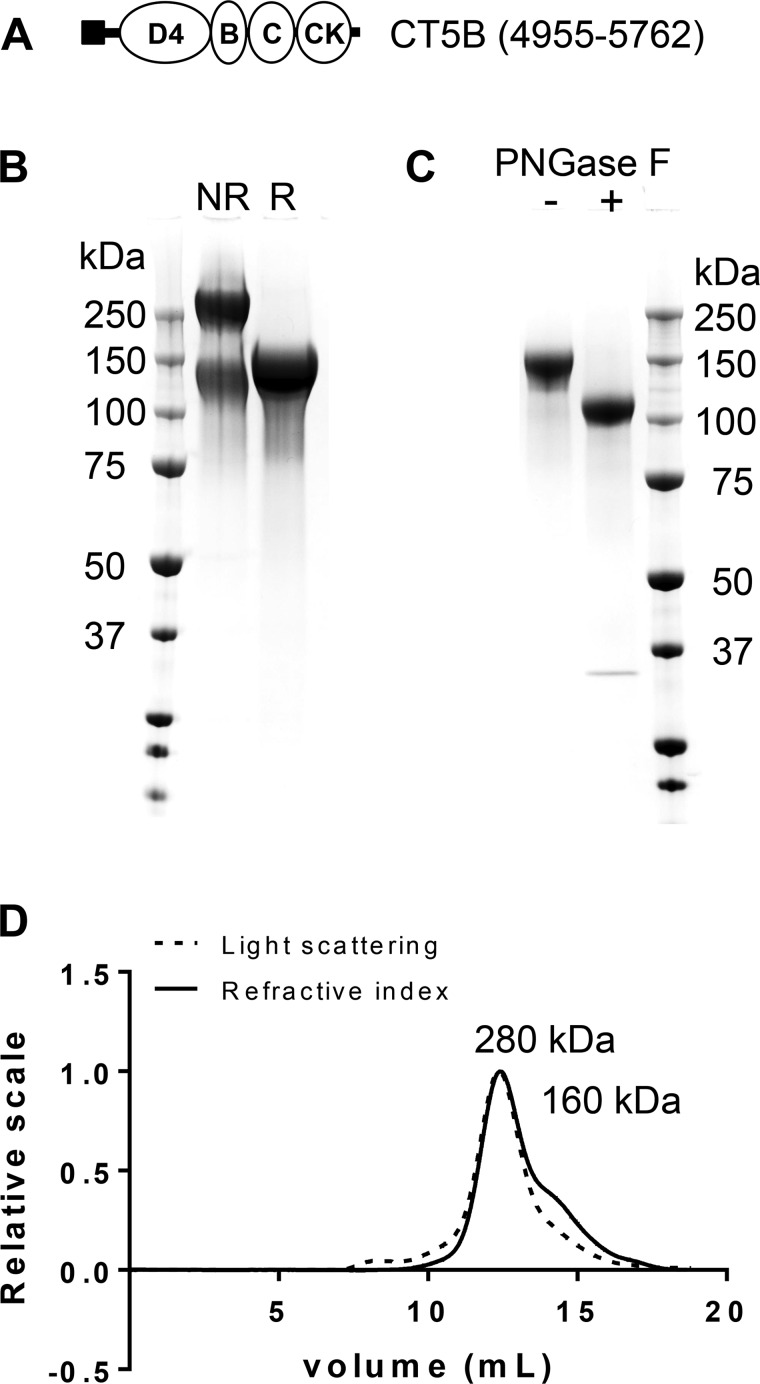
Expression of recombinant MUC5B COOH-terminal protein domain. *A*: schematic showing the domains of the COOH-terminal construct [CT5B; D4-B-C-CK domains (open ovals), His-Tag (solid square)] of MUC5B. *B*: the expressed COOH-terminal construct was analyzed by SDS-PAGE [reduced (R) and nonreduced (NR)] and stained with InstantBlue. Different CT5B protein preparations yielded varying proportions of monomer and dimer. *C*: reduced CT5B was incubated with (+) or without (−) PNGase F and analyzed by SDS-PAGE and stained with InstantBlue. *D*: SEC-MALLS analysis of CT5B showing light scattering (dashed line) and refractive index (solid line); molecular mass values of CT5B monomer and dimer are shown.

A MUC5B (UniProtKB accession number Q9HC84) COOH-terminal construct was created consisting of D4-B-C-CK (CT5B; residues 4955 and 5762; Fig. 2*A*) (4). The recombinant protein was expressed with an NH_2_-terminal His6 tag using the mammalian episomal expression vector pCEP-His in 293-EBNA (19). Conditioned medium was collected from stably transfected 293-EBNA cells and analyzed by SDS-PAGE. Recombinant protein was purified by nickel-affinity chromatography followed by size exclusion (Superose 6) and anion-exchange chromatography (Resource Q) as described previously (18). Size exclusion chromatography multiangle laser light scattering (SEC-MALLS) was performed as described previously to determine the molecular size of the expressed protein (18). CT5B was treated with PNGase F (NEB, Hitchin, UK) under denaturing conditions following the manufacturers' protocol.

#### Analysis of mucin cleavage by density gradient centrifugation.

Density gradient centrifugation was performed on HBEC and CF BEC apical secretions in a Beckman Ti70 rotor, at 40,000 rpm 68 h 15°C, in 4 M GdmCl/CsCl at a starting density of 1.4 g/ml. Tubes were unloaded from the top and fractions were analyzed for glycoproteins with periodic acid-Schiff (PAS) staining and MUC5B by use of immunodetection after slot blotting onto nitrocellulose (31). Mucin-containing fractions were pooled and then reduced with 10 mM DTT for 1 h at 37°C, followed by carboxymethylation using 25 mM iodoacetamide and incubation at room temperature in the dark for 15 min. The reduced sample was subjected to a second isopycnic centrifugation with the same conditions, fractions were analyzed as above, and MUC5B-containing fractions were pooled. Tandem mass spectrometry (MS) was performed on the pooled mucin containing fractions (unreduced mucins were reduced and carboxymethylated as above). In brief, samples were dialyzed into water to remove GdmCl and CsCl, lyophilized, and resuspended in 0.2 M urea-0.1 M ammonium bicarbonate prior to trypsin digestion overnight at 37°C. Samples were applied to a Superdex 100 column to separate peptides from higher molecular weight glycopeptides. Peptides were desalted by using ZipTip (EMD Millipore, Billerica, MA) prior to separation on a nanoAcquity liquid chromatography system (Waters, Milford, MA) before being automatically analyzed on an LTQ Velos ion trap mass spectrometer (Thermo Scientific, Waltham, MA) as previously described (21). Data were analyzed with Mascot software (Matrix Science, Boston, MA) against a human International Protein Index database.

#### Neutrophil elastase treatment of MUC5B.

MUC5B (100 μg/ml) was incubated with or without 1 μg/ml neutrophil elastase (6) in PBS for 4 or 24 h at 37°C and then reduced and carboxymethylated as above. The sample was buffer exchanged to give final concentration 0.5 M GdmCl/0.1 M ammonium bicarbonate by use of a Vivaspin 100-kDa MWCO filter and then digested with trypsin and analyzed by tandem MS (see above).

#### Antibodies.

MUC5AC [polyclonal antiserum MAN-5ACI (anti-*O*-glycosylated and non-*O*-glycosylated MUC5AC) (27)] was used at 1:2,000 for immuno- and Western blots and monoclonal antibody Mab2011 (anti-non-*O*-glycosylated MUC5AC) (25) was used at 1:2,000 for immuno- and Western blots; MUC5B [polyclonal antiserum MAN-5BI (anti-*O*-glycosylated and non-*O*-glycosylated MUC5B) (29)] was used at 1:2,000 for immuno- and Western blots; and polyclonal antiserum LUM5B-13 (anti-non-*O*-glycosylated MUC5B, provided by Ingemar Carlstedt, Lund University, Lund, Sweden) raised against the synthetic peptide TPSSTPGTTWILTC was used at 1:1,000 for immuno- and Western blots. Band intensities were quantitated by use of Quantity One software and GS-800 scanner (Bio-Rad Laboratories, Hercules, CA).

## RESULTS

### 

#### Identification of intracellular molecular forms of MUC5B.

A series of experiments was performed to characterize the intracellular forms of MUC5B produced by the HBECs grown in ALI culture. For the initial studies, cell lysates were subjected to agarose gel electrophoresis and subsequent Western blots were analyzed with MUC5B-specific probes. LUM5B-13, raised against a peptide sequence that is masked in *O*-glycosylated MUC5B, specifically recognizes the non-*O*-glycosylated mucin (Fig. 1*A*). The other antiserum, MAN-5BI, raised against a peptide sequence in the Cys-domains that interrupt the main glycosylated portion of MUC5B, recognizes both non-*O*-glycosylated and *O*-glycosylated MUC5B (Fig. 1*A*). Unreduced, non-*O*-glycosylated MUC5B migrated as two bands with high electrophoretic mobility (Fig. 1*B*; *lane 1*). After reduction, a single band was observed that migrated further into the gel (Fig. 1*B*; *lane 5*). The non-*O*-glycosylated forms of MUC5B likely represent *1*) a disulfide-linked multimer, probably native polypeptide dimer (Fig. 1*B*; *lane 1*, annotated as NPD), *2*) native polypeptide monomer (Fig. 1*B*; *lane 1*, annotated NPM); and *3*) reduced polypeptide monomer (Fig. 1*B*; *lane 5*, annotated as RPM). Importantly, the probe for non-*O*-glycosylated MUC5B identified bands that were distinct from those detected by Mab2011 (Fig. 1*B*; *lanes 2* and *6*), a monoclonal antibody specific for non-*O*-glycosylated MUC5AC, the other polymeric mucin produced by these cells in culture (28).

In contrast to the non-*O*-glycosylated forms of MUC5B, *O*-glycosylated, polymeric MUC5B had a markedly lower electrophoretic mobility and barely migrated into the gel (Fig. 1*B*; *lane 3*, annotated as OGP). After treatment with DTT, the resultant reduced, *O*-glycosylated monomer exhibited a marked increase in electrophoretic mobility (Fig. 1*B*; *lane 7*, annotated as ROGM). Neither native nor reduced *O*-glycosylated mucins were reactive with LUM5B-13, the non-*O*-glycosylated mucin probe (Fig. 1*B*; *lanes 1* and *5*), even after overexposure of the blots. Furthermore, under normal conditions, none of the MUC5B in the apical secretions from the cells was reactive with LUM5B-13 (data not shown). It is noteworthy that the *O*-glycosylated MUC5B is indistinguishable by electrophoresis from MUC5AC, either at the polymeric mucin level (Fig. 1*B*; compare *lanes 3* and *4*) or as reduced *O*-glycosylated monomers (Fig. 1*B*; compare *lanes 7* and *8*).

To further substantiate the assignments of the different intracellular forms of MUC5B, HBEC lysates were analyzed by CsCl/4 M GdmCl density gradient centrifugation (Fig. 1*C*). This technique was previously shown to separate *O*-glycosylated mucins (high buoyant density), from non-*O*-glycosylated mucins (low buoyant density) (1, 25). Slot blotting of fractions across the density gradient with LUM5B-13 and MAN-5BI showed the separation of *O*-glycosylated MUC5B (1.38–1.5 g/ml) from the non-*O*-glycosylated polypeptide (1.25–1.32 g/ml) (Fig. 1*C*).

In summary, Western blotting of the cell lysate (Fig. 1*B*) and immunoblotting of the density gradient fractions (Fig. 1*C*) demonstrated that MAN-5BI most strongly detected the *O*-glycosylated form of MUC5B, whereas LUM-5B13 detected only the polypeptide precursors. The MAN-5BI data indicated that *O*-glycosylated MUC5B was the major form of the mucin within the cells. Furthermore, it is important to note that MUC5B is the predominant mucin produced by this culture model (2, 8, 28).

#### The COOH-terminal region of MUC5B (CT5B) forms homotypic disulfide-linked dimers.

Non-*O*-glycosylated dimers are proposed to be the first step in mucin polymer formation and are suggested to be formed by covalent disulfide linkage between MUC5B COOH-termini (18, 33); however, it has not been definitively shown that the COOH-terminal region of MUC5B can form dimers. To address this issue, a recombinant protein encompassing the MUC5B COOH-terminal region (D4-B-C-CK domains; Fig. 2*A*) was expressed in 293-EBNA cells and purified. The construct was secreted by the 293-EBNA cells and the identity of purified protein was confirmed by tandem MS. Purified recombinant protein was analyzed by SDS-PAGE (Fig. 2*B*) and by SEC-MALLS (Fig. 2*D*). SDS-PAGE showed that the apparent molecular size of the expressed CT5B monomer is higher than that expected from its polypeptide sequence (∼150 kDa compared with ∼90 kDa); removal of *N*-glycans using PNGase F showed that *N*-glycosylation accounted for a major part of the difference (Fig. 2*C*). Both SDS-PAGE and SEC-MALLS showed that CT5B was present as disulfide-stabilized dimers and monomers. These results showed that the CT5B domain expressed in 293-EBNA cells replicated the intermolecular disulfide links observed in intact MUC5B.

#### Time scale of MUC5B biosynthesis and secretion of newly synthesized polymers.

Agarose gel electrophoresis, which we have demonstrated separates non-*O*-glycosylated from *O*-glycosylated MUC5B, was used in combination with pulse radiolabeling to investigate MUC5B synthesis. In the following experiments [^35^S]methionine was used to metabolically label newly synthesized MUC5B in HBECs studied at baseline, i.e., in the absence of exogenous secretagogue (37). Preliminary experiments established that culture of cells for 4 h in methionine-free media, prior to addition of the pulse of radiolabeled amino acid, resulted in sufficient ^35^S-incorporation to monitor mucin biosynthesis.

#### Non-O-glycosylated MUC5B synthesis.

**Fig. 3.**
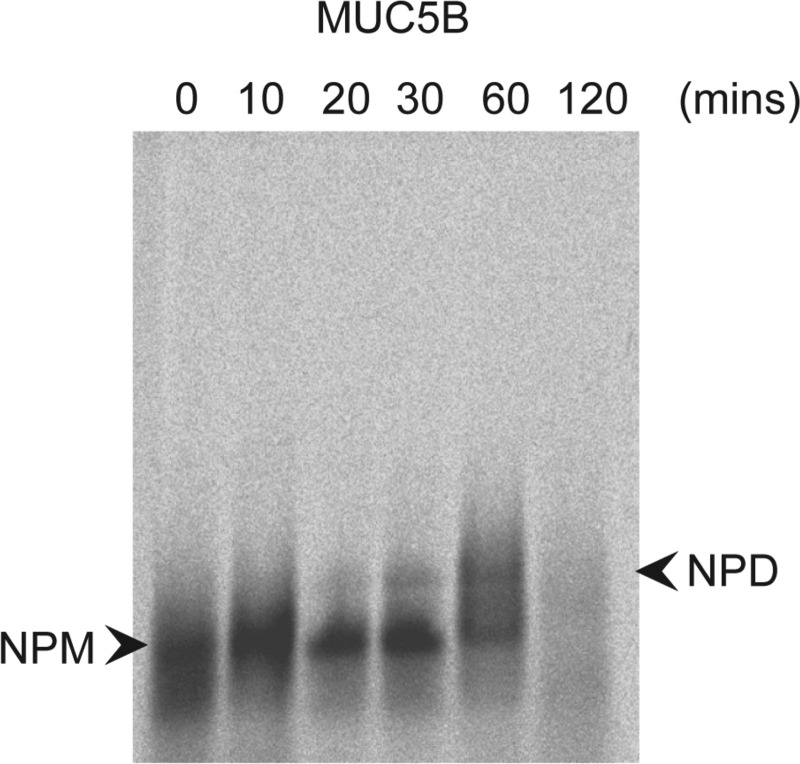
Time course of non-*O*-glycosylated mucin polypeptide synthesis. HBECs were pulse labeled for 15 min with [^35^S]methionine and chased for 0, 10, 20, 30, 60, and 120 min. Non-*O*-glycosylated molecular intermediates were immunoprecipitated from cell lysates with LUM-5B13. Immunoprecipitates were subjected to agarose gel electrophoresis and after electrophoresis the gel was treated with 10 mM DTT to reduce the mucins, which were then transferred to nitrocellulose by vacuum blotting prior to detection of incorporated radioactivity by phosphoimaging. NPM, native polypeptide monomer; NPD, native polypeptide dimer.

A pulse-chase experiment was performed to ascertain the time course of non-*O*-glycosylated MUC5B synthesis. After a 15-min pulse-label with [^35^S]methionine the cells were lysed, at various chase times (0, 10, 20, 30, 60, and 120 min), and the lysates were subjected to immunoprecipitation with LUM5B-13. Agarose gel electrophoresis of the immunoprecipitates showed transfer of radioactivity from non-*O*-glycosylated monomers (NPM) to dimers (NPD) over the 2 h chase period (Fig. 3). After 1 h of chase non-*O*-glycosylated MUC5B was more fully converted from NPM to putative NPD. Non-*O*-glycosylated MUC5B had almost totally disappeared within 2 h postlabel. As already shown in Fig. 1*B*, both radiolabeled NPM and putative NPD were sensitive to reduction (data not shown).

#### O-glycosylated MUC5B synthesis.

**Fig. 4.**
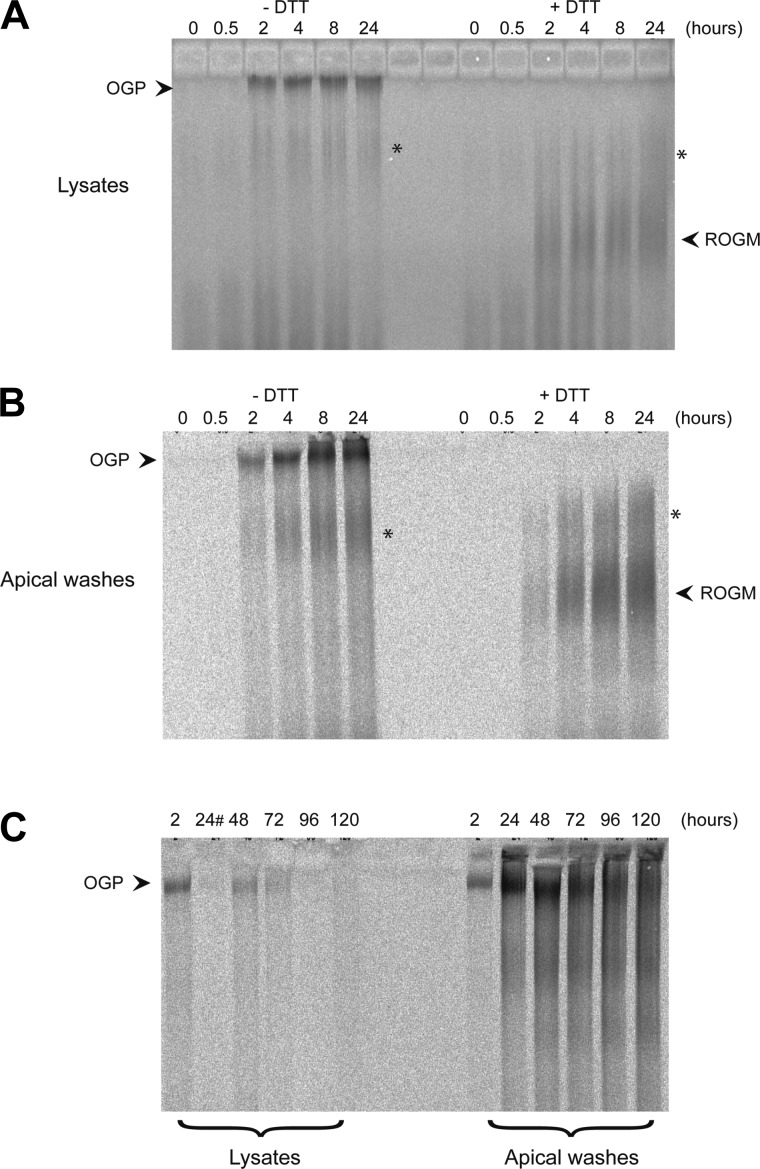
Time course of *O*-glycosylated polymeric mucin synthesis. *A* and *B*: HBECs were pulse-labeled for 20 min with [^35^S]methionine and chased for 0, 0.5, 2, 4, 8, and 24 h. Apical washes and cell lysates (native and treated with 10 mM DTT) at each time point were subjected to agarose gel electrophoresis. After electrophoresis the gel was treated with 10 mM DTT to reduce the mucins, which were then transferred to nitrocellulose by vacuum blotting prior to detection of incorporated radioactivity by phosphoimaging. *A*: native (left half of gel) and reduced (right half of gel) cell lysates. *B*: native (left half of gel) and reduced (right half of gel) apical washes. *C*: a pulse-chase experiment performed as above except that HBECs were pulse labeled for 30 min with [^35^S]methionine and chased for a longer time (24, 72, 96, and 120 h). In *C*, the cell lysates are on the left half of the gel and the apical washes on the right half. #The well for the 24-h cell lysate time point leaked. Inspection of the gels shows a smeared radioactive band (highlighted with an asterisk) in the cell lysates and apical washes that was insensitive to reduction. Previous analysis of HBE cultures suggests this might represent the nonpolymeric mucins MUC1, MUC4, and MUC16 (3).

Further pulse-chase studies were performed to analyze the synthesis of *O*-glycosylated MUC5B, as well as the time course of its apical secretion. After a 20-min pulse-label with [^35^S]methionine the intracellular (Fig. 4*A*, left-side lanes), and secreted (Fig. 4*B*, left-side lanes) forms of MUC5B were analyzed by agarose gel electrophoresis (at various chase times: 0, 0.5, 2, 4, 8, and 24 h). A band consistent with the electrophoretic migration of *O*-glycosylated, polymeric MUC5B (see Fig. 1*B*; *lane 3*) was visible inside the cells after 2 h postlabel (Fig. 4*A*) and remained throughout the experiment (up to 24 h). *O*-glycosylated, polymeric MUC5B was secreted within 2 h postlabel (Fig. 4*B*) and the radioactivity associated with the secreted polymeric mucins increased to a maximum after 8 h. To verify the assignment of these radiolabeled bands as polymeric mucins the samples were treated with DTT prior to electrophoresis. This resulted in the disappearance of the slow migrating radiolabeled bands and the appearance of faster migrating smeared bands (Fig. 4, *A* and *B*, right-side lanes). This change in mobility, after treatment with DTT, is consistent with the change in electrophoretic properties of polymeric *O*-glycosylated MUC5B shown in Fig. 1*B*. Furthermore, the major radioactive bands (before and after reduction) were reactive with MAN-5BI, but not with the non-*O*-glycosylated mucin polypeptide probe LUM5B-13 (data not shown). Taken together these data confirmed that the radiolabeled molecules found at the top of the gel were *O*-glycosylated mucin polymers. However, it is important to reiterate that we cannot distinguish between MUC5B and the other polymeric mucin produced by these cells, MUC5AC, since both mucins had similar electrophoretic properties and neither of the mucin antisera (MAN-5BI and MAN-5ACI) were able to quantitatively immunoprecipitate the *O*-glycosylated mucins.

To investigate the fate of the radiolabeled *O*-glycosylated polymers over a longer time scale, the experiment was repeated with increased chase times (48, 72, 96, and 120 h). The results showed that the majority of the radiolabeled mucins were released from the cells at baseline by 72–96 h (Fig. 4*C*). The loss of radioactivity inside the cells (Fig. 4*C*, left-side lanes) was mirrored by the accumulation of radiolabeled polymers in the apical secretions (Fig. 4*C*, right-side lanes). With increasing time in the apical secretion, the radioactivity associated with the largest mucin polymers decreased and a ladder pattern emerged, suggesting that the mucins had undergone a degree of extracellular depolymerization due to proteolytic degradation or disruption of disulfide linkages.

#### Is MUC5B proteolytically processed during biogenesis?

**Fig. 5.**
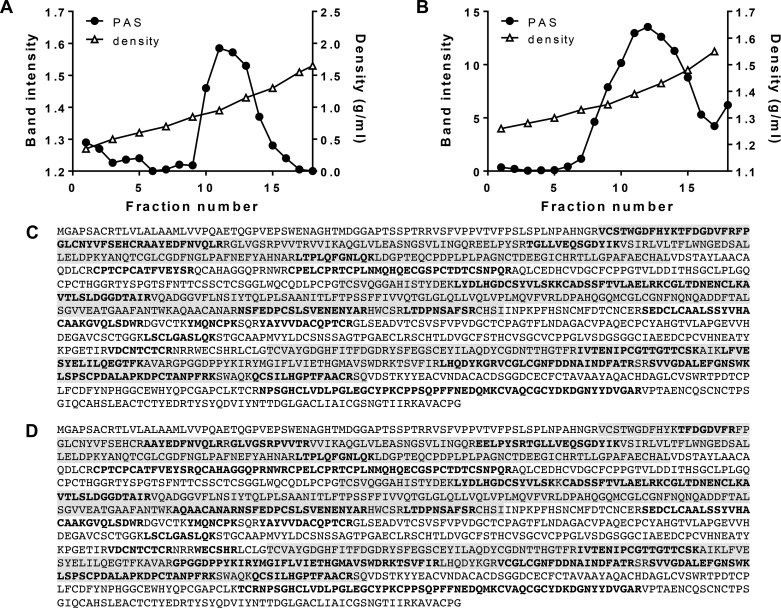
Analysis of proteolytic cleavage of MUC5B secreted from HBECs in air-liquid interface culture. *A*: unreduced cell wash samples were subjected to isopycnic CsCl density gradient centrifugation in 4 M GdmCl with a starting density of 1.4 g/ml. After slot blotting onto nitrocellulose the *O*-glycosylated MUC5B distribution was analyzed by PAS staining (●). Fractions were also analyzed for density by weighing (△). *B*: fractions (10–16) containing high-density *O*-glycosylated MUC5B from *A* were reduced, carboxymethylated, and subjected to a further CsCl density gradient in 0.2 M GdmCl with a starting density of 1.5 g/ml. After slot blotting onto nitrocellulose the *O*-glycosylated MUC5B distribution was analyzed by PAS staining (●). *C*: tryptic peptides generated from the high-density MUC5B peak (fractions 7–18) in *B* were analyzed by tandem mass spectrometry. The 32 peptides that matched the NH_2_-terminus of the human MUC5B sequence (accession number Q9HC84) are highlighted in bold text. The shaded highlighting identifies the D1, D2, and D3 domains. *D*: 36 tryptic peptides were generated from MUC5B in the apical secretions from HBECs stimulated by the application of 100 μM ATPγS (37).

Proteolytic cleavage by furin at the NH_2_-terminus of the related polymeric glycoprotein vWF is required for its packing into secretory granules; the cleaved propeptide (DI-D2) remains associated with the mature polymer, via a calcium-mediated interaction with the D3 domain, until after secretion when the change in environment (increased pH and decreased Ca^2+^) cause it to dissociate and the polymer unfurls (23, 34). Unlike vWF, MUC5B lacks the NH_2_-terminal furin-cleavage site; nevertheless, NH_2_-terminal proteolytic cleavage has been suggested to play a role in the postsecretory expansion of MUC5B (11). To assess whether proteolytic processing removes sections of the NH_2_-terminus of MUC5B during biosynthesis and secretion, we analyzed mucins produced by HBECs cultured at the ALI. Apical secretions from the cells were subjected to 4 M GdmCl/CsCl density gradient centrifugation (Fig. 5*A*) to separate mucin polymers from proteolytically processed D-domains. The low-buoyant density fractions (fractions 1–6; expected to contain nonmucin proteins together with potential protein-rich fragments released from mucins) and the high-buoyant density *O*-glycosylated mucin-rich fraction (fractions 10–16) were subjected to trypsin digestion followed by tandem MS to identify the major proteins present. There were no peptides identified from MUC5B in the low buoyant density fraction. In contrast, in the high-buoyant density mucin-rich fraction, 48 peptides were identified from MUC5B, which were distributed throughout the molecule (data not shown). These results showed no evidence for the absence of NH_2_- (or COOH)-terminal protein domains from MUC5B and suggested that, if the polypeptide of MUC5B had been cleaved, then the fragment(s) had remained attached to the polymer. This is not unlikely because the terminal regions of MUC5B contain multiple intramolecular disulfide linkages and polypeptide cleavage will not necessarily generate “free” fragments. However, this possibility was ruled out by subsequent experiments in which the mucins were reduced and carboxymethylated and resubjected to CsCl/4 M GdmCl density gradient centrifugation (Fig. 5*B*). Again, tandem MS analysis did not reveal any MUC5B peptides in low-buoyant density fractions (*fractions 1*-*5*) and analysis of the high-density reduced mucin fraction (*fractions 7*-*18*) revealed 32 peptides across the NH_2_-terminus of the MUC5B polypeptide (Fig. 5*C*). Tandem MS analysis of MUC5B produced by ATP-stimulation of HBECs revealed a similar distribution of tryptic peptides across the NH_2_-terminus of the polypeptide (Fig. 5*D*). These two sets of tandem MS data indicate that MUC5B is not proteolytically processed during biogenesis.

#### Is MUC5B proteolytically processed during biogenesis in CF airway cells?

**Fig. 6.**
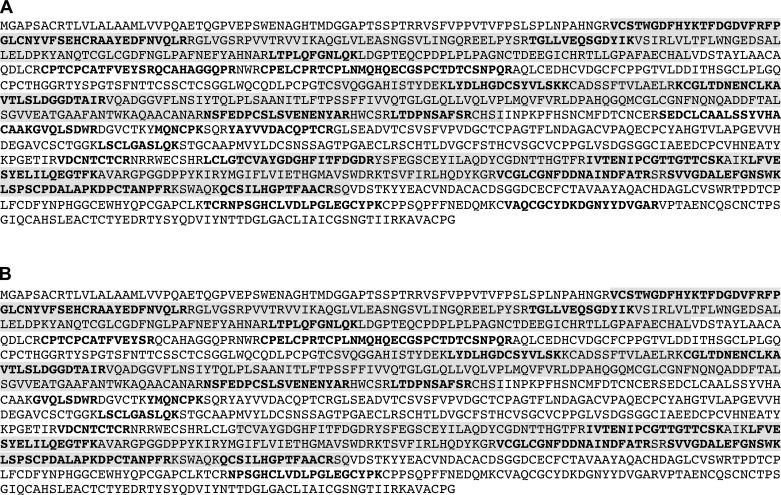
Analysis of proteolytic cleavage of MUC5B in the cell lysates and apical secretions from CF BECs in air-liquid interface culture. Reduced and carboxymethylated MUC5B mucins were isolated following the two-step isopycnic CsCl density gradient centrifugation. Tryptic peptides generated from the high-density reduced and carboxymethylated MUC5B from the second density gradient (0.2 M GdmCl) of the cell lysate (*A*) and the apical secretions (*B*) were analyzed by tandem mass spectrometry. The peptides that matched the NH_2_-terminus of the human MUC5B sequence (accession number Q9HC84) are highlighted in bold text. The shaded highlighting identifies the D1, D2, and D3 domains.

Proteolytic cleavage of MUC5B has been reported in CF (6, 9); to assess whether this occurs during synthesis, we employed the tryptic peptide mapping approach outlined to analyze glycosylated MUC5B isolated by CsCl density gradient centrifugation (by the 2-step approach shown in Fig. 5) of cell lysates or apical secretions from CF airway cells cultured at ALI (data not shown). Tandem MS analysis of the reduced and carboxymethylated high-buoyant density glycosylated MUC5B from both cell lysates and apical secretions revealed peptides across the NH_2_-terminus of the MUC5B polypeptide (Fig. 6). These results show that, as in normal HBECs, the MUC5B polypeptide is not proteolytically processed in CF BECs during its biogenesis or in the apical secretions, at least under sterile conditions. To analyze the effect of a protease found in CF sputum on MUC5B, we treated MUC5B with neutrophil elastase [1 μg/ml (6) for 4 and 24 h]. Tandem MS analysis of elastase-treated MUC5B showed a reduction in NH_2_-terminal peptides (21 unique peptides at 4 h; 18 unique peptides at 24 h) compared with untreated MUC5B (29 unique peptides at 4 h; 34 unique peptides at 24 h). These results support that proteolytic cleavage of MUC5B reported in CF is an extracellular event in sputum.

## DISCUSSION

MUC5B is a major polymeric mucin in airway mucus (13). Here we have shown that, under baseline conditions, which represents the predominant mode of mucin secretion (37) polymeric MUC5B is assembled within 2 h by primary airway cells in culture. After this time a proportion of MUC5B is secreted from the apical surface of the cells while the majority of MUC5B is retained within the cells and released over the next 48 h. The time scale for MUC5B intracellular assembly is in agreement with previous findings on MUC5B in gall bladder tissue explants, which reported secretion of newly synthesized MUC5B after 4 h (32). It is noteworthy that the other major airway mucin, MUC5AC, shows a similar time course of intracellular assembly (25).

We were able to distinguish on basis of buoyant density and electrophoretic mobility between *O*-glycosylated and non-*O*-glycosylated MUC5B. Using this approach we have shown that *O*-glycosylated MUC5B is the major form of the mucin within the cells and, importantly, that *O*-glycosylated MUC5B (not non-*O*-glycosylated) is secreted from cells. Thus *O*-glycosylation of MUC5B is likely to be a prerequisite for passage through the secretory pathway; however, experimental verification is necessary to test the observation.

Previous studies have shown that MUC5B follows a similar intracellular assembly to the related polymeric glycoprotein, vWF (18), although not all steps have been formally demonstrated. Here we add to the knowledge of the assembly process by showing that MUC5B polymerization occurs through COOH-terminal dimerization, mediated via disulfide linkage, which precedes subsequent NH_2_-terminal multimerization via disulfide linkage between D3 domains (18). Similar to vWF and MUC5AC, MUC5B forms linear polymers (11, 18).

An important step in vWF biogenesis is furin cleavage at the NH_2_-terminus, which facilitates a pH-dependent, noncovalent calcium interaction between the cleaved vWF propeptide (DI-D2) and disulfide-linked D3 dimers that has been proposed to organize vWF polymers for storage within Weibel-Palade bodies (10). Reports in the literature, based on Western blotting, have suggested that MUC5B also undergoes NH_2_-terminal proteolytic cleavage (35, 36). However, the MS-based approach employed herein provides no evidence for a similar cleavage in MUC5B liberating a DI-D2 fragment, under steady-state and stimulated conditions. Further supporting this finding is the lack of a similar furin cleavage site in the MUC5B polypeptide. Therefore, our data highlight important differences in the intracellular assembly and packaging of vWF and MUC5B. Moreover, our data demonstrate that proteolytic cleavage is not an obligate step in the assembly and packaging mechanism of MUC5B. Indeed, our recent study suggested that only the intact NH_2_-terminal domain of MUC5B is involved in pH-and calcium-dependent interaction that promotes ordered packing of the polymer within the secretory granule (18). Maybe this difference in assembly is not too surprising considering that MUC5B contains much larger central glycosylated domains, as indicated by the monomer molecular masses of vWF, ∼360 kDa, and of MUC5B, 2.0–2.5 MDa. The five- to sevenfold larger size of the mucins may dictate a different intragranular organization.

Regarding the intragranular organization of MUC5B, recent work has shown that MUC5B is highly organized within the secretory granule with mucin chains organized around proteinaceous nodes. Upon release from the granule the MUC5B mucin changes macromolecular structure from a condensed to an expanded form (11). Kesimer and coworkers (11) have proposed that one factor controlling the postsecretory expansion of MUC5B is proteolytic cleavage. Our data do not support a proteolytic cleavage within the MUC5B D-domains; however, it is quite possible that cleavage of as yet unidentified, accessory “packaging” proteins in the nodes drive the mucin expansion process. More complete analysis is required of the proteinaceous nodes that appear to form focal link points organizing the mucin within the granule.

MUC5B and the other major airway polymeric mucin, MUC5AC, isolated from CF sputum show extensive evidence of proteolytic degradation (6, 9). Our data provide strong evidence that proteolytic cleavage of MUC5B is not part of its biogenesis in CF airway cells. Furthermore, unlike in CF sputum, MUC5B is not degraded in the apical secretions from CF BECs in culture. Thus in CF sputum, MUC5B degradation arises from bacterial or host inflammatory cell proteases (7), likely compromising its protective function. Indeed, we have confirmed that a CF-related protease, neutrophil elastase, does modify the NH_2_-terminal region of MUC5B.

In summary, we have demonstrated that the major airway mucin MUC5B in a physiologically relevant model of the airway epithelium follows a similar molecular assembly to vWF with the exception of NH_2_-terminal proteolytic cleavage, suggesting that the intragranular organization of MUC5B polymers is likely to differ from vWF. Future work is needed to define the molecular details of MUC5B organization within the secretory granules and the mechanisms of its postsecretory expansion. Interestingly, it has recently been reported in a murine model of CF that intestinal goblet cell secretory granules are more alkaline pH than wild-type mice goblet cell secretory granules and this results in defective mucin exocytosis (14). One might speculate that the aberrant mucin exocytosis is caused by disruption of the calcium-dependent pH organization of mucins within secretory granules that is active at acidic pH (18) and that alkalization of the granules could negatively impact mucus formation in the intestine, lung, and other mucus-producing organs.

## GRANTS

This work was supported by grants from Cystic Fibrosis Foundation Therapeutics (THORNT07XXX0) and The Wellcome Trust (065833/Z/01/Z). The Wellcome Trust Centre for Cell-Matrix Research, University of Manchester, is supported by core funding from the Wellcome Trust [088785/Z/09/Z].

## DISCLOSURES

No conflicts of interest, financial or otherwise, are declared by the author(s).

## AUTHOR CONTRIBUTIONS

C.R., S.K., and S.J.W. performed experiments; C.R. and S.K. prepared figures; C.R., S.K., and D.J.T. drafted manuscript; C.R., C.W.D., P.W., and D.J.T. edited and revised manuscript; C.R. and D.J.T. approved final version of manuscript; S.K., S.J.W., and D.J.T. analyzed data; C.W.D., P.W., and D.J.T. interpreted results of experiments; P.W. and D.J.T. conception and design of research.
